# POEMS Syndrome: A Case Report and Review of the Literature

**DOI:** 10.7759/cureus.27001

**Published:** 2022-07-19

**Authors:** Deepthi Kanuganti, Venkata Sundarachary Nagarjunakonda, Pranathi Bandarupalli, Vamsi Krishna Gorijala, Venkata Lakshman Sai Ram Konagalla, Praveen Kowtha

**Affiliations:** 1 Neurology, Dr. Ramesh Cardiac and Multispeciality Hospital, Guntur, IND; 2 Neurology, Guntur Medical College, Guntur, IND; 3 Neurology, Alluri Sitaramaraju Academy of Medical Sciences, Eluru, IND; 4 Neurology, Katuri Medical College and Hospital, Guntur, IND; 5 Internal Medicine, R-Endocrinology, Hamilton, USA

**Keywords:** plasma cell dyscrasia, systemic autoimmune disease, castleman variant of poems syndrome, castleman disease, poems syndrome

## Abstract

Polyneuropathy, organomegaly, endocrinopathy, monoclonal protein elevation, and skin changes (POEMS) syndrome is a rare multisystem disorder that occurs due to an underlying plasma cell dyscrasia. A diagnosis is made with the presence of two mandatory criteria and at least one major and one minor criterion. We present a case of a 28-year-old patient who presented with weakness of bilateral arms and legs, thinning of hands, and swelling of bilateral lower limbs and abdomen. The patient also reported weight loss and loss of appetite. Examination revealed areflexic quadriparesis with sensory loss, diffuse lymphadenopathy, pleural effusion, ascites, and pulmonary hypertension. Investigations showed elevated erythrocyte sedimentation rate (ESR). Nerve conduction studies revealed severe axonal polyneuropathy of all nerves. Lymph node biopsy showed Castleman disease. A diagnosis of POEMS syndrome was made and he was sent for a stem cell transplant, which is the definitive treatment in patients eligible for stem cell transplant.

## Introduction

Polyneuropathy, organomegaly, endocrinopathy, monoclonal protein elevation, and skin changes (POEMS) syndrome is a rare paraneoplastic disorder. However not included in the acronym include other important features like ascites, papilledema, pleural effusion, Castleman disease, etc. It was first described by Crow in 1956 and then by Fukase in 1968 [1]. The acronym "POEMS" was coined by Bardwick et al. in 1980 [2]. It is mostly seen in the 60s with a slight male predominance [1]. POEMS syndrome is associated with an excessive plasma cell proliferation [2]. Owing to its rarity, it is usually underdiagnosed unless included in the differential. The median survival rate is eight to 14 years without successful treatment [3]. Neither a single symptom or a single test can establish the diagnosis of POEMS syndrome. Diagnosis of POEMS syndrome needs two mandatory criteria, major and minor criteria that we have discussed in detail below [4]. POEMS syndrome is associated with a rise in pro-inflammatory cytokines [4]. The severity of the disease activity correlates with the levels of vascular endothelial growth factor (VEGF) [4]. We describe a rare case of a young patient who presented classically with all the criteria necessary for the diagnosis of POEMS syndrome.

## Case presentation

A 28-year-old male patient with no past medical history presented with weakness of bilateral arms and legs, thinning of hands, swelling of bilateral lower limbs, and abdominal distension with protruding umbilicus for six months. He also complained of breathlessness for the past six months. He noticed that the weakness started initially in the distal muscles of the lower limbs and gradually involved the proximal muscles of the lower limbs. Later, both proximal and distal muscles of the upper limbs were involved. There was associated wasting and deformity of the hands. The patient had swelling of bilateral lower limbs up to the level of thighs. There was distension of the abdomen with protrusion of umbilicus and edema over the groin area. He had shortness of breath of grade 2-3 severity. He noticed a weight loss of 6-8 kg and a loss of appetite for the last year. The patient denied sensory complaints, involuntary movements, fever, jaundice, chest pain, and hemoptysis.

**Figure 1:**
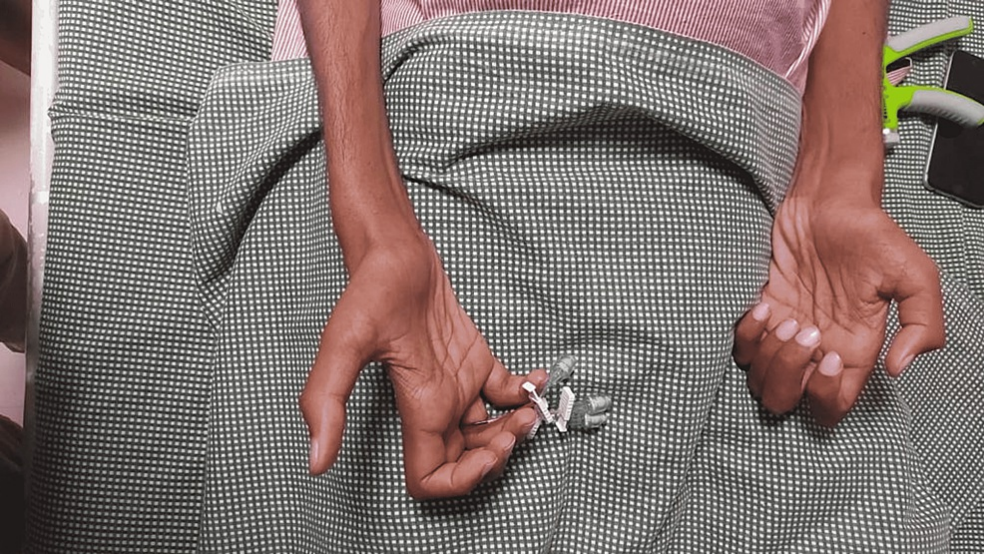
Bilateral claw hand with wasting of intrinsic muscles.

General examination revealed non-pitting edema of both lower limbs with dry and thickened skin. There was inguinal, axillary, and cervical lymphadenopathy. Neurologic examination showed areflexic quadriparesis with glove and stocking sensory loss, bilateral claw hand, and foot drop (Figure 1). There was no cranial nerve involvement. Ophthalmic examination was negative for papilledema. Higher mental functions were normal. Cardiovascular examination showed tachycardia. Respiratory system examination revealed tachypnea and decreased breath sounds in the bilateral lower zones. Gastrointestinal system examination revealed ascites and umbilical hernia.

Investigations at the time of admission showed hemoglobin of 9.8 mg/dL (reference range {N}: 12-16 mg/dL), platelet count of 5.04x10^5^/cu mm (N: 1.5-4 10^5^/cu mm), and erythrocyte sedimentation rate (ESR) of 72 mm/h (N: ≤15 mm/h) with normal peripheral smear. Thyroid function tests revealed T3 of 58.9 ng/dL (N: 80-220 ng/dL), T4 of 4.32 mcg/dL (N: 5-12 mcg/dL), and thyroid-stimulating hormone (TSH) of 13.6 mIU/L (N: 0.4-4 mIU/L). Urinalysis showed <500 mg of protein/24 h.

**Figure 2:**
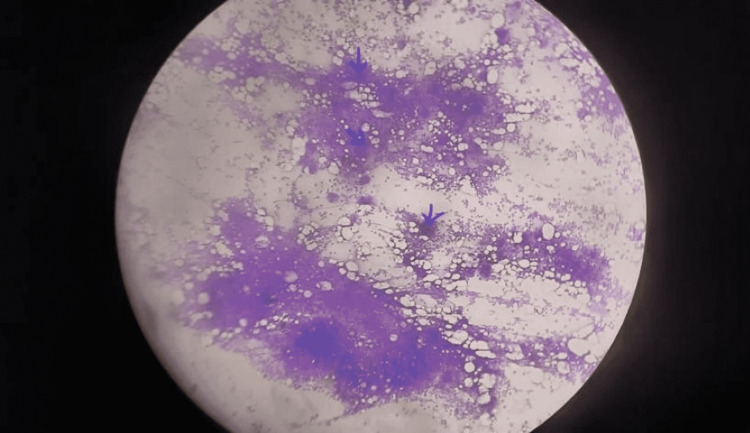
Bone marrow biopsy showing marked megakaryocytosis.

**Figure 3:**
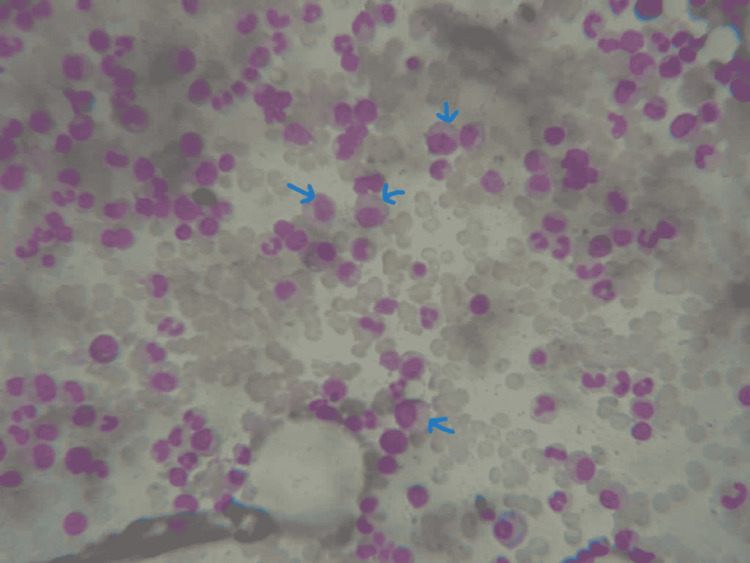
Bone marrow biopsy showing increased plasma cells (N <10%). N: reference range

An ultrasound of the abdomen showed moderate hepatosplenomegaly, ascites, and inguinal lymphadenopathy. A chest x-ray revealed bilateral pleural effusion. Echocardiography showed pericardial effusion with a tricuspid regurgitation gradient (TRG) of 46 mmHg (N: <36 mmHg). Nerve conduction velocities were suggestive of severe axonal polyneuropathy of all the nerves. CSF analysis showed albuminocytological dissociation with CSF cell count of 1/cu mm (N: 0-5 cells/cu mm) and CSF protein of 117 mg/dL (N: 15-45 mg/dL). Ascitic fluid analysis showed a transudate with serum-ascites albumin gradient (SAAG) of <1.1 g/dL. CT screening for sclerotic bone lesions was negative for bone lesions. Bone marrow biopsy revealed megakaryocytic hyperplasia (Figure 2) and plasmacytosis with 10% (N: <10%) plasma cells (Figure 3).

**Figure 4:**
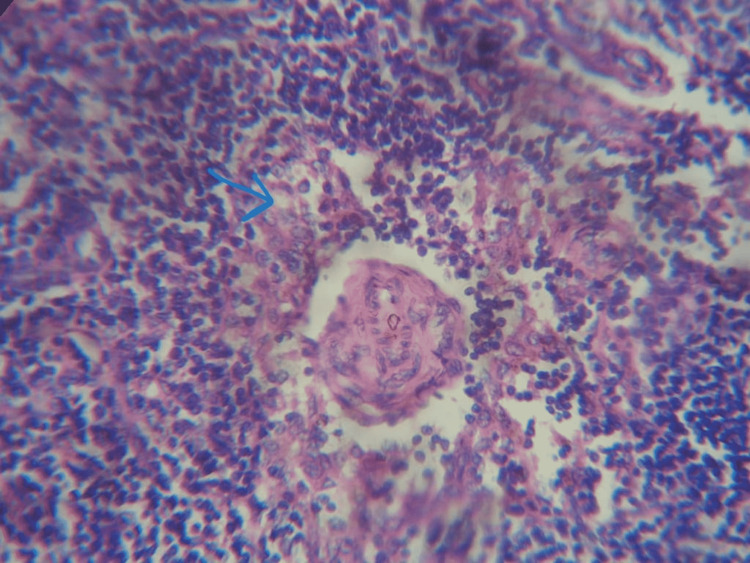
Lymph node biopsy showing marked vascular proliferation (Castleman disease).

Lymph node biopsy was performed and reported to have a highly vascular lymph node disease, Castleman disease (Figure 4). He was found to have elevated myeloma (M) protein lambda chain on serum protein electrophoresis. Serum immunofixation electrophoresis (IFE) showed immunoglobulin G lambda monoclonal protein. After aligning clinical features with investigations, the diagnosis of POEMS syndrome was made. The patient was administered 1 g of intravenous methylprednisolone. He was referred to a higher center for stem cell transplantation.

## Discussion

POEMS syndrome is a rare paraneoplastic syndrome due to an underlying plasma cell dyscrasia. The pathophysiology of POEMS is poorly understood. It is an autoinflammatory disease that is thought to occur due to the presence of a monoclonal plasma cell population and a coexisting rise in pro-inflammatory cytokines like interleukin-6 (IL-6), IL-1β, and tumor necrosis factor-alpha (TNF-α) [4]. These plasma cells are lambda chain restricted in most cases [5]. There is restricted usage of immunoglobulin lambda light chain variable region (IGLV) genes derived from IGLV 1-40 to 1-44 [6]. Various cytogenetic abnormalities have been associated with the causation and prognosis of POEMS syndrome: aneuploidy including monosomy 13 and trisomy 3 and 7 [7], and 14q32 translocations and 13q14 deletion have also been described [8].

The most commonly increased cytokine is VEGF. In POEMS syndrome patients, VEGF levels correlate with disease activity and also estimate treatment response and survival [9,10]. The diagnosis of POEMS syndrome can be made when patients meet the two mandatory criteria and at least one major and one minor criterion (Table 1).

**Table 1:** Diagnostic criteria for POEMS syndrome. The table is adapted from Dispenzieri (2019) [5]. POEMS: polyneuropathy, organomegaly, endocrinopathy, monoclonal protein elevation, and skin changes; VEGF: vascular endothelial growth factor

Mandatory criteria	Polyneuropathy and monoclonal plasma cell proliferative disorder
Other major criteria	Castleman disease or angiofollicular lymph node hyperplasia, osteosclerotic lesions, elevated serum or plasma VEGF levels.
Minor criteria	Organomegaly (splenomegaly, hepatomegaly, or lymphadenopathy), extravascular volume overload (peripheral edema, ascites, or pleural effusion), endocrinopathy (adrenal, thyroid, pituitary, gonadal, parathyroid, or pancreatic), skin changes (hyperpigmentation, hypertrichosis, glomeruloid hemangiomata, plethora, acrocyanosis, flushing, white nails), papilledema, thrombocytosis or polycythemia.

Castleman disease is a non-clonal lymphoproliferative disorder that involves heterogeneous proliferation of lymphocytes [11]. It is seen in about 11-30% of patients diagnosed with POEMS syndrome [11]. It is frequently linked to HIV and human herpesvirus-8 (HHV-8) [11]. The pathophysiology of Castleman disease is associated with an increase in inflammatory cytokines most commonly, interleukin-6 (IL-6) by B-lymphocytes in the germinal centers of lymph nodes. Increased IL-6 causes an increase in B-lymphocytes and plasma cell growth, which contributes to tumor proliferation and constitutional B symptoms [12]. There is an increase in VEGF levels, which increases the vascularity of tumor progression [12]. When Castleman is diagnosed in a patient with a clinical picture suggestive of POEMS syndrome, but without evidence of neuropathy or monoclonal protein, it is referred to as the Castleman variant of POEMS syndrome [13]. This case report is unique because POEMS syndrome, diagnosed in our patient, was seen in a young individual, in contrast to the older population, where it is normally prevalent. Our patient demonstrated both the mandatory criteria. His lymph node biopsy showed Castleman disease, meeting one of the major criteria. He also satisfied several minor criteria like organomegaly, extravascular volume overload, endocrinopathy, and thrombocytosis.

Certain diseases such as chronic inflammatory demyelinating polyneuropathy (CIDP), multiple myeloma, monoclonal gammopathy of undetermined significance (MGUS) neuropathy, and light chain amyloidopathy mimic POEMS syndrome [13]. It is very important to differentiate between them and make a correct diagnosis as treatment modalities and the expected treatment-related toxicities are quite different for each of the above conditions [5].

Treatment of POEMS syndrome is determined by the extent of bone marrow involvement as evident by blind iliac crest sampling [5]. In cases with isolated bone lesions (≤3 lesions) without clonal plasma cells, radiation therapy is recommended as the first-line therapy and has been shown to attain a 10-year survival rate of 70% [14]. In case of disseminated bone marrow involvement, systemic therapy is recommended [15]. In cases with large bony lesions with a significant lytic component, adjuvant or neoadjuvant radiation therapy can be considered along with chemotherapy [5]. High-dose melphalan plus autologous stem cell transplant (ASCT), melphalan plus dexamethasone, and lenalidomide plus dexamethasone are the most effective systemic therapies [5,16-18]. Other promising therapies include thalidomide plus dexamethasone and bortezomib plus dexamethasone which can be used as a potential therapy for those ineligible for ASCT [19,20]. There are a few case reports that highlighted the success of bortezomib in the treatment of POEMS syndrome [21-23]. Some case reports reported limited success with interferon-alpha, tamoxifen, trans-retinoic acid, ticlopidine, argatroban, and strontium-89 [24]. Supportive therapy like physical therapy, continuous positive airway pressure (CPAP), analgesics such as gabapentin and tramadol for neuropathic pain, and psychological consultation and orthotics play an important role in the recovery of patients [5]. Hormone replacement therapy with thyroxine or prednisone provides clinical benefits to patients with hypothyroidism or adrenal insufficiency [11]. We need to conduct large-scale randomized studies to come up with a treatment strategy to treat this multisystem disorder.

Treatment response for POEMS can be monitored with plasma VEGF levels and the concentration of M proteins in the blood every three to four months [13]. All the therapies produce some degree of neurological improvement which can be assessed with the Neuropathy Limitation Scale [13]. The use of systemic therapy in patients should be followed up with right ventricular systolic pressure (echocardiogram), and diffusing capacity for carbon monoxide (DLCO) every six months [5]. Positron emission tomography (PET)/CT is beneficial to identify relapses. It is advisable to follow up with the patient on a quarterly basis to track the above parameters after therapy induction [5].

The course of POEMS syndrome is chronic and overall survival has improved over the years with advancements in treatment strategies like the advent of stem cell transplantation and immunomodulatory therapies. The 10-year survival rates for patients diagnosed before and after 2003 were 55% and 79%, respectively [14]. Favorable prognostic factors include albumin >3.2 g/dL, attainment of complete hematologic response, and younger age at diagnosis [5]. Wang et al. developed and validated a risk nomogram that predicts long-term outcomes in POEMS syndrome patients [25]. They described some poor prognostic factors, including age greater than 50 years, presence of pleural effusion at the time of presentation, severe renal impairment i.e., estimated glomerular filtration rate (eGFR) <30 mL/min/1.73 m^2^, and pulmonary hypertension [25]. Other poor prognostic factors include clubbing of fingernails, edema, ascites, respiratory symptoms, impaired DLCO, and papilledema [5]. Notably, the survival rate is not affected by the number of POEMS features present [5].

## Conclusions

POEMS syndrome is a rare multisystem disorder that has high morbidity and mortality if left untreated. It is usually seen in patients in their sixties. We presented a case report of a 28-year-old patient with classic POEMS syndrome symptoms. Early diagnosis and treatment can prevent irreversible damage. There are multiple treatment options that are currently being used around the world. Large-scale clinical trials are needed to identify the most effective treatment option.
